# Honey-Conjugated Honeybee Brood Biopeptides Improve Gastrointestinal Stability, Antioxidant Capacity, and Alleviate Diet-Induced Metabolic Syndrome in a Rat Model

**DOI:** 10.3390/foods14162907

**Published:** 2025-08-21

**Authors:** Sakaewan Ounjaijean, Supakit Chaipoot, Rewat Phongphisutthinant, Gochakorn Kanthakat, Sirinya Taya, Pattavara Pathomrungsiyounggul, Pairote Wiriyacharee, Kongsak Boonyapranai

**Affiliations:** 1 Research Institute for Health Sciences, Chiang Mai University, Chiang Mai 50200, Thailand; sakaewan.o@cmu.ac.th; 2 Multidisciplinary Research Institute, Chiang Mai University, Chiang Mai 50200, Thailand; supakit.ch@cmu.ac.th (S.C.); rewat.psn@gmail.com (R.P.); pairote.w@cmu.ac.th (P.W.); 3 Research Center of Microbial Diversity and Sustainable Utilization, Faculty of Science, Chiang Mai University, Chiang Mai 50200, Thailand; 4 Faculty of Agro-Industry, Chiang Mai University, Chiang Mai 50100, Thailand; gochakorn.g@gmail.com (G.K.); pattavara.p@cmu.ac.th (P.P.); 5 Processing and Product Development Factory, The Royal Project Foundation, Chiang Mai 50100, Thailand

**Keywords:** honeybee brood biopeptides, Maillard reaction, gastrointestinal stability, antioxidant activity, metabolic syndrome

## Abstract

Honeybee brood biopeptides (HBb-Bps) are a novel source of bioactive compounds with potential health benefits. In this study, HBb-Bps were conjugated with honey via a Maillard reaction and their physicochemical properties, digestive stability, antioxidant capacity, and anti-obesogenic effects were evaluated. Simulated gastrointestinal digestion revealed significantly enhanced resistance after conjugation, with the residual content increasing from 46.99% for native HBb-Bps to 86.12% for the honey-conjugated forms; furthermore, antioxidant activity was largely preserved according to the DPPH and ABTS assays. In the in vivo experiments, 30 male BrlHan: WIST@Jcl (GALAS) (Wistar) rats were fed a high-fat diet (HFD) to induce obesity and orally administered honey-conjugated HBb-Bps at doses of 200, 500, or 1000 mg/kg body weight for 16 weeks. The highest dose led to significant reductions in body weight gain, the Lee index, and body mass index. The serum lipid profiles markedly improved, with decreases in the total cholesterol, triglyceride, and LDL levels, as well as cardiovascular risk indices. Furthermore, fecal analysis showed increased levels of short-chain fatty acids, particularly butyrate. These changes suggest enhanced gut microbial activity; however, the prebiotic effects were inferred from the SCFA profiles, as the gut microbiota composition was not directly analyzed. In conclusion, honey-conjugated HBb-Bps improve gastrointestinal stability and exhibit antioxidant, hypolipidemic, and gut-modulating effects, supporting their potential use as functional ingredients for managing diet-induced metabolic disorders.

## 1. Introduction

Protein hydrolysates and their resulting biopeptides have garnered increasing attention as functional food ingredients due to their wide-ranging biological activities, including antioxidant, antihypertensive, immunomodulatory, and prebiotic effects. Produced through enzymatic or microbial hydrolysis, these peptides exhibit enhanced bioavailability and may serve as modulators of gut health and systemic metabolic functions [1,2,3,4,5].

Among the emerging sources of biopeptides, honeybee brood (*Apis* spp.) represents a novel and sustainable insect-derived protein resource [6,7]. Our previous research has demonstrated that enzymatically and microbially hydrolyzed honeybee brood proteins, or honeybee brood biopeptides (HBb-Bps), possess strong antioxidant activity and potential prebiotic properties [8]. In particular, HBb-Bps are conjugated with reducing sugars such as glucose, fructose, or honey via the Maillard reaction, these conjugates exhibit enhanced bio-functionality—most notably, the ability to support the growth of beneficial gut bacteria, including selected probiotic strains [9]. This work further demonstrated that honey-conjugated HBb-Bps exhibited greater antioxidant activity than conjugates prepared with individual sugars such as glucose or fructose, likely due to the synergistic effects of honey’s diverse constituents and multiple Maillard reaction pathways. These findings suggest that HBb-Bps and their honey-derived conjugates could be promising candidates for the development of next-generation synbiotic formulations.

In this study, honey was chosen as the Maillard conjugate partner due to its superior functional enhancement and complex composition beyond simple sugars. While fructose and glucose constitute approximately 70–80% of honey, honey also contains organic acids, amino acids, minerals, enzymes, flavonoids, and phenolic compounds [10]. Its complex biochemical environment favors multiple glycation pathways, enabling the formation of Maillard reaction products, including melanoidins, that exhibit enhanced stability and antioxidant features [11]. Furthermore, honey’s non-digestible oligosaccharides have been increasingly recognized for their prebiotic potential, selectively promoting beneficial gut microbes [12]. These synergistic attributes make honey a promising substrate for peptide conjugation, with the potential to improve gastrointestinal resilience and metabolic health; however, its role in enhancing peptide stability and function remains underexplored.

Biopeptides have been reported to support gut health by promoting the growth of beneficial probiotic strains and by modulating gut microbial metabolites such as short-chain fatty acids (SCFAs), which are closely associated with intestinal and systemic health [13,14]. Furthermore, several studies have indicated that dietary peptides may contribute to the regulation of metabolic parameters, including lipid profiles and glucose homeostasis, particularly in models of diet-induced obesity [15]. However, despite these promising observations, several key challenges remain. Many food-derived peptides are susceptible to enzymatic degradation in the upper gastrointestinal (GI) tract, which can limit their bioavailability and functional efficacy [16]. While glycation through the Maillard reaction has been proposed as a method to enhance the structural stability and functionality of peptides [17], few studies have explored whether such conjugation can protect peptides from digestive breakdown and simultaneously enhance their bioactivities in rat models.

To address these gaps, this study aimed to evaluate the functional potential of honey-conjugated honeybee brood biopeptides, with a focus on their digestive stability, antioxidant activity, and effects on lipid profiles, adiposity, and short-chain fatty acid production in a high-fat diet-induced rat model. We hypothesized that conjugating honeybee brood biopeptides with honey via the Maillard reaction would improve their resistance to gastrointestinal digestion, preserve their antioxidant capacity, and enhance their prebiotic functionality. We further anticipated that these improvements would lead to beneficial effects on metabolic health.

## 2. Materials and Methods

### 2.1. Materials and Chemicals

Honeybee brood (*Apis mellifera* L.) was harvested from a Longan honey farm located in Maewang District, Chiang Mai Province, Thailand. The raw bee brood was steamed using a stainless-steel steamer for 1 h and then stored in plastic bags in a freezer until further use. The raw longan honey used in the study was purchased from Koonton Bee Farm in Sansai District, Chiang Mai Province, Thailand. The strain of *Rhizopus oligosporus* TISTR3527 (*R. oligosporus*) was obtained from the Biodiversity Research Centre (BRC) of the Thailand Institute of Scientific and Technological Research (TISTR) in Pathum Thani, Thailand. All chemicals and enzymes were purchased from Sigma-Aldrich (Burlington, MA, USA).

### 2.2. Preparation of Honey-Conjugated HBb-Bps Using the Moist–Dry Heating Method with Spontaneous Aging Technique

The honeybee brood biopeptides (HBb-Bps) used in this study were prepared following the method described by Ounjaijean et al. (2024) [9], which involves alkaline protein extraction, defatting, and fungal fermentation using *R. oligosporus* under solid-state conditions. The HBb-Bps were further subjected to conjugation with honey via the Maillard reaction, employing a moist–dry heating method adapted from a previous study [8]. Briefly, a 1% (*w*/*v*) HBb-Bps solution was prepared in deionized water and mixed at a 1:1 (*v*/*v*) ratio with 1% (*w*/*v*) solutions of honey. The resulting mixtures were incubated in a desiccator maintained at 75% relative humidity and 60 °C for 20 days to induce the Maillard reaction. Upon completion of the reaction period, the samples were immediately frozen at –18 °C to terminate the reaction and stored for subsequent biological characterization.

### 2.3. In Vitro Simulated Gastrointestinal Digestion of HBb-Bps Conjugated with Honey

The in vitro gastrointestinal digestion of the HBb-Bps and honey-conjugated HBb-Bps was performed using a standardized static digestion model adapted from Minekus et al. [18] and Monente et al. [19], with slight modifications. The protocol involved three sequential phases: oral, gastric, and intestinal digestion. For the oral phase, 2.5 g of a lyophilized sample was mixed with 17.5 mL of Simulated Salivary Fluid (SSF), 1.5 mL of an α-amylase (A3176, Sigma-Aldrich) solution (1.3 mg/mL), 125 µL of 0.3 M CaCl_2_, and deionized water to a final volume of 25 mL. The mixture was incubated at 37 °C for 30 min under dark conditions. Subsequently, the gastric phase was initiated by adding 18.75 mL of Simulated Gastric Fluid (SGF), 1.86 mL of a pepsin solution (prepared by dissolving 1 g of pepsin (P7125, Sigma-Aldrich) in 10 mL of 0.1 M HCl), 12.5 µL of 0.3 M CaCl_2_, and deionized water up to 25 mL; the pH was adjusted to 3.0 with 1 M HCl. The samples were incubated at 37 °C for 2 h in darkness. In the intestinal phase, 27.5 mL of Simulated Intestinal Fluid (SIF), 12.5 mL of a pancreatin (P7545, Sigma-Aldrich) solution (0.008 g/mL), 6.25 mL of a bile salt solution (0.025 g/mL), 100 µL of 0.3 M CaCl_2_, and deionized water up to a final volume of 50 mL were added. The pH was adjusted to 7.0 using 1 M NaOH. The digestion was carried out at 37 °C in a shaking water bath throughout the process.

To evaluate the extent of carbohydrate hydrolysis and protein degradation resulting from enzymatic digestion during the different gastrointestinal phases, samples were collected at the end of each digestion stage (oral, gastric, and intestinal). The reducing sugar content was quantified using the CheKine™ Micro Reducing Sugar (RS) Assay Kit (MyBioSource, Inc., San Diego, CA, USA) while the concentration of primary amino nitrogen was determined using the Primary Amino Nitrogen Assay Kit (Megazyme, Sydney, Australia), according to the manufacturers’ protocols. The degree of hydrolysis (DH%) was calculated based on the combined amount of reducing sugars and free amino acids released during digestion relative to the initial sample weight according to the following formula:

DH (%) = ((C_RS_ + C_FAA_) × 100)/W_initial_ (1)

where C_RS_ = content of reducing sugars released (mg); C_FAA_ = content of free amino acids released (mg); and W_initial_ = initial dry weight of the sample (mg).

### 2.4. Antioxidant Activities of HBb-Bps Conjugated with Honey

The antioxidant activities of the HBb-Bps and honey-conjugated HBb-Bps were assessed using two independent assays—the DPPH and ABTS radical-scavenging assays—using established protocols. For the DPPH assay, a 0.1 mM DPPH solution in methanol was prepared and mixed with the sample at a ratio of 2.4 mL of the DPPH solution to 1.6 mL of the sample solution. The mixture was vortexed and incubated in the dark at ambient temperature for 30 min. Absorbance was subsequently measured at 517 nm using a spectrophotometer. Trolox was employed to construct a standard calibration curve, and the antioxidant capacity of each sample was expressed as the percentage of DPPH radical production inhibited.

The antioxidant potential of the samples was also evaluated using the ABTS radical cation decolorization assay, following a previously reported method with minor modifications [20]. To generate the ABTS^+^• radical, an oxidizing mixture of 7 mM ABTS and 2.45 mM potassium persulfate in 20 mM sodium acetate buffer (pH 4.5) was prepared and incubated at room temperature in the dark for 12–16 h. The resulting radical solution was then diluted with 95% ethanol to achieve an absorbance of 0.70 ± 0.02 at 734 nm; this solution served as the working solution. Subsequently, 100 µL of each sample was added to 3 mL of the ABTS^+^• working solution, and the mixture was incubated at 30 °C for 10 min in darkness. The absorbance was recorded at 734 nm, and the percentage inhibition was calculated using a Trolox standard curve to express the antioxidant capacity.

### 2.5. Animal Experiments

A total of 30 male BrlHan: WIST@Jcl (GALAS) (Wistar) rats, weighing 180–200 g, were obtained from Nomura Siam International Co., Ltd. The animals were housed in cages, with three in each cage, at 25 ± 2 °C and with a 12 h light/dark cycle. They were acclimatized for one week before the experimental procedures were performed. All experimental protocols received approval from the Animal Ethics Committee, Laboratory Animal Center, Chiang Mai University (2567/RT-0012), and followed the institutional guidelines for the Care and Use of Laboratory Animals. The rats were sustained on a commercial balanced diet. The animals were randomly assigned to the experimental groups using a computer-generated randomization schedule after one week of acclimatization. Randomization was stratified to ensure comparable baseline body weights across the groups. Outcome assessments were conducted by laboratory personnel who were blinded to the treatment allocation to minimize bias. The group coding was only revealed after the data collection and statistical analyses were completed. The animals were randomly divided into five groups (*n* = 6 per group). Group 1, the normal diet control group (ND), received a standard chow diet containing 4.02 kcal/g (20% fat, 28% protein, and 52% carbohydrate). Groups 2–5 were fed a high-fat diet (HFD; 5.35 kcal/g, 52% fat, 20% protein, and 28% carbohydrate) for 16 weeks to induce obesity. Group 2 served as the HFD control and received distilled water as the vehicle-only control, while groups 3–5 were designated as treatment groups and received the test compound (honey-conjugated HBb-Bps) via oral gavage at doses of 200, 500, and 1000 mg/kg body weight, respectively, once daily throughout the study period. Body weight and food intake were monitored regularly during the experiment.

At the end of the 16-week period, the final body weight and body length were recorded to calculate the Lee Index using the following formula:

Lee Index = (∛body weight in grams/body length in cm) × 1000(2)

Following an overnight fast, all the animals were anesthetized by intraperitoneal (i.p.) injection of pentobarbital sodium (150 mg/kg). Blood was collected via cardiac puncture using both heparinized and EDTA-coated tubes for the subsequent biochemical analyses. After humane euthanasia, a necropsy was performed to excise and weigh the internal organs. Any visible abnormalities or discoloration were noted. Fecal samples were also collected and stored for the determination of short-chain fatty acid (SCFA) levels.

### 2.6. Detection of Biochemical Indexes in Rat Serum

The collected blood samples were centrifuged to obtain serum for biochemical analysis. The levels of total cholesterol (TC), triglycerides (TG), high-density lipoprotein cholesterol (HDL-C), and low-density lipoprotein cholesterol (LDL-C) were measured using an automated analyzer (Randox Laboratories, Crumlin, UK) following the manufacturer’s instructions. Based on these measurements, the Atherogenic Index (AI) was calculated using the formula

AI = (TC − HDL-C)/HDL-C,(3)

while the Cardiac Risk Factor (CR) was determined as

CR = TC/HDL-C.(4)

These indices were used to assess lipid metabolism and cardiovascular risk associated with the dietary intervention and test compound administration.

### 2.7. Short-Chain Fatty Acid (SCFA) Measurement

Approximately 0.5 g of fecal material was suspended in 5 mL of distilled water and vigorously vortexed for 5 min to ensure thorough homogenization. The resulting mixture was acidified to a pH of 2–3 using a 5 M hydrochloric acid solution. After acid adjustment, the samples were agitated for an additional 10 min, followed by centrifugation at 5000 rpm for 20 min. The supernatant was transferred into chromatographic vials for subsequent analysis. The concentrations of short-chain fatty acids (SCFAs), including acetate, propionate, and butyrate, were quantified following the method described by Alvarez et al. [21], utilizing high-performance liquid chromatography (HPLC) equipped with a refractive index detector (RID-20A, Shimadzu, Kyoto, Japan). Separation was achieved using an Aminex HPX-87H column (300 mm × 7.8 mm; Bio-Rad, Hercules, CA, USA) maintained at 50 °C. The mobile phase consisted of 3 mM sulfuric acid, operated under isocratic conditions with a flow rate of 0.6 mL/min.

### 2.8. Statistical Analysis

The results are presented as the mean + standard derivation (SD) obtained from three independent experiments. Data distribution normality was assessed using the Shapiro–Wilk test prior to applying parametric tests. Statistical analyses were performed using one-way ANOVA with the SPSS 22 statistical software (Chicago, IL, USA). Post hoc comparisons were conducted using Tukey’s test to determine significant differences between groups. Exact *p*-values and effect sizes (Hedges’ *g*) with 95% confidence intervals were calculated for all pairwise comparisons. *p* < 0.05 was considered statistically significant.

## 3. Results

### 3.1. In Vitro Simulated Gastrointestinal Digestion of HBb-Bps and Honey-Conjugated HBb-Bps

The simulated gastrointestinal digestion revealed distinct differences in the hydrolysis patterns and residual inulin, HBb-Bp, and honey-conjugated HBb-Bp contents (Table 1). Inulin exhibited minimal hydrolysis across all digestive phases, with values of 0.04 ± 0.01% in the oral phase, 1.60 ± 0.23% in the gastric phase, and 15.85 ± 1.27% in the intestinal phase. Its high residual post-digestion content (82.51 ± 3.14%) reflects its strong resistance to enzymatic degradation, consistent with its known prebiotic nature. In contrast, the HBb-Bps demonstrated pronounced hydrolysis, particularly in the gastric phase (36.41 ± 2.14%), indicating sensitivity to acidic and proteolytic conditions. Hydrolysis was also observed in the oral (4.13 ± 0.34%) and intestinal (12.46 ± 1.18%) phases, resulting in a markedly lower residual content (46.99 ± 2.44%) compared to inulin. Interestingly, the honey-conjugated HBb-Bps showed significantly enhanced resistance to digestion, particularly in the oral and gastric phases, with hydrolysis values of only 0.27 ± 0.08% and 0.36 ± 0.10%, respectively. Despite moderate hydrolysis in the intestinal phase (13.25 ± 3.14%), the overall residual content remained high at 86.12 ± 4.01%, surpassing the inulin and the unmodified HBb-Bp contents. These findings suggest that honey conjugation enhances the structural stability of HBb-Bps, potentially protecting the peptides from premature degradation and allowing for targeted release in the lower gastrointestinal tract.

Data are expressed as mean ± standard deviation (SD) from three independent experiments (*n* = 3). Residual Post-Digestion Content (%) refers to the percentage of peptide mass remaining after simulated gastrointestinal digestion, calculated relative to the initial peptide content.

**Figure 1:**
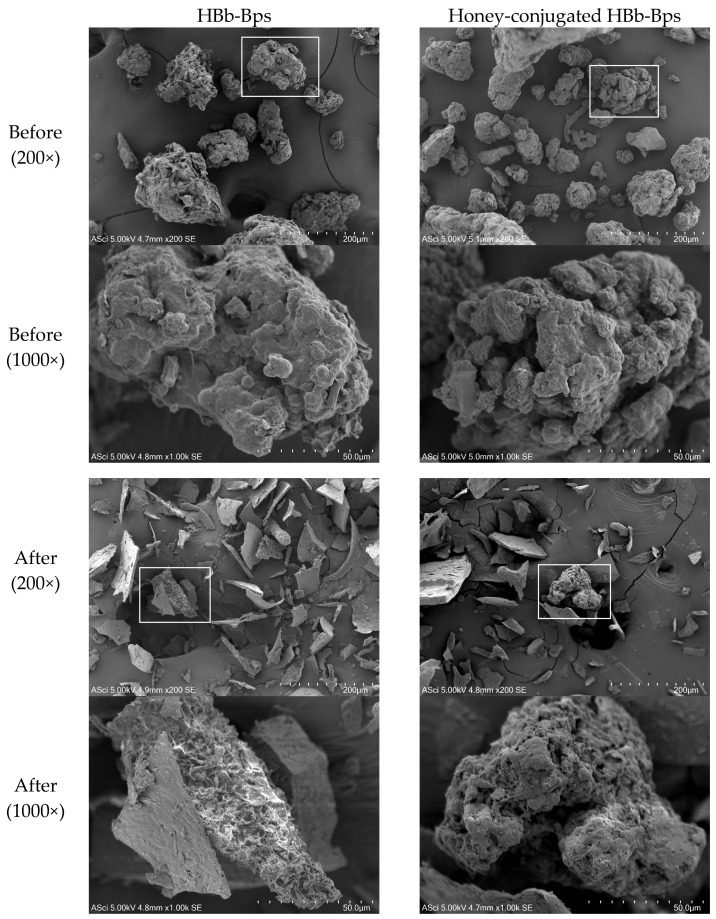
Scanning electron micrographs of the HBb-Bps and honey-conjugated HBb-Bps before and after the Maillard reaction at 200× and 1000× magnification. The lower panels (1000×) represent higher magnification images of the regions indicated by the white boxes in the corresponding 200× images, providing detailed visualization of surface morphology and particle aggregation.

As shown in Figure 1, scanning electron microscopy (SEM) revealed distinct differences in the surface morphology of the HBb-Bps and honey-conjugated HBb-Bps before and after the Maillard reaction. The native HBb-Bps (upper left) displayed irregular, aggregated structures with rough and uneven surfaces, while the post-reaction HBb-Bps (lower left) exhibited thinner, more fragmented, and brittle-like particles, indicating structural breakdown during the digestive process. In contrast, the honey-conjugated HBb-Bps (upper right) showed more compact and cohesive particle formation, suggesting enhanced molecular interactions and stabilization due to honey incorporation. Following the Maillard reaction (lower right), the conjugated particles became more porous and sponge-like, possibly as a result of melanoidin formation and complex cross-linking between the peptides and honey constituents. These structural modifications may contribute to an improved moisture-holding capacity and digestion resistance, consistent with the higher residual content observed after the simulated gastrointestinal digestion. Overall, the SEM analysis supports the hypothesis that honey conjugation alters the microstructure of HBb-Bps in a way that enhances their functional and physicochemical stability.

### 3.2. Antioxidant Activities of HBb-Bps Conjugated with Honey Before and After in Vitro Simulated Gastrointestinal Digestion

The antioxidant capacity of the HBb-Bps and honey-conjugated HBb-Bps was assessed using DPPH and ABTS radical-scavenging assays, both prior to and following simulated gastrointestinal digestion. These assays were employed to determine the extent to which honey conjugation influences the HBb-Bps’ free radical-scavenging activity and the stability of their antioxidant properties under digestive conditions. As shown in Table 2, the honey-conjugated HBb-Bps consistently exhibited significantly higher antioxidant activity than the unmodified HBb-Bps in both assays. For the DPPH, pre-digestion activity increased from 31.04 ± 2.41% in HBb-Bps to 43.28 ± 4.10% in the conjugates (*p* = 0.0021), a large effect size (Hedges’ g = 2.91), indicating a substantial practical difference. Post-digestion, the activity of both groups declined, but the conjugates retained markedly higher activity (25.61 ± 2.39% vs. 15.36 ± 3.45%, *p* = 0.0054) with a similarly large effect size (g = 2.76), suggesting that honey conjugation provides strong protection against antioxidant loss during digestion. A similar pattern was observed in the ABTS assay. Before digestion, conjugated peptides showed higher scavenging activity (70.54 ± 3.48%) compared to HBb-Bps (54.21 ± 5.34%, *p* = 0.0315, g = 2.90). After digestion, the conjugates maintained a markedly superior activity (60.32 ± 4.27% vs. 32.14 ± 4.25%, *p* = 0.0104) with an exceptionally large effect size (g = 5.90), underscoring their enhanced stability and bioactivity under gastrointestinal conditions. These findings indicate that the Maillard-type conjugation with honey substantially improves and preserves the antioxidant properties of HBb-Bps during digestion, likely due to the formation of stable, bioactive compounds such as melanoidins.

### 3.3. Effect of Honey-Conjugated HBb-Bps on High-Fat Diet-Induced Metabolic Syndrome in Rats

**Figure 2:**
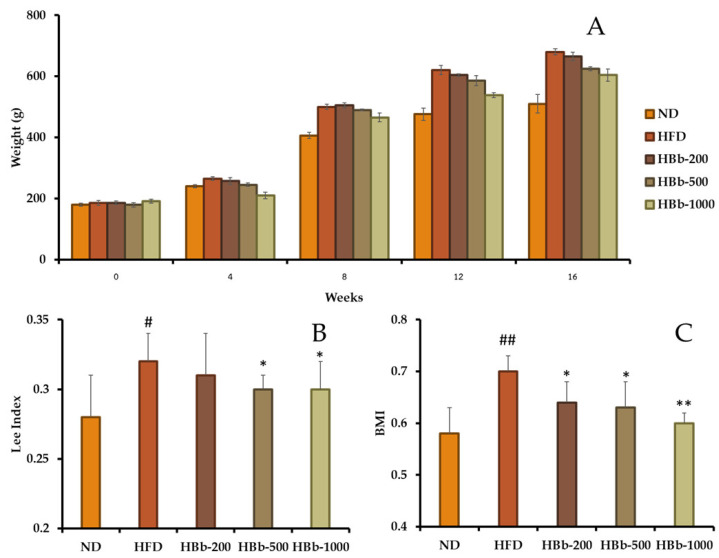
Effects of honey-conjugated HBb-Bps on body weight, Lee Index, and BMI in Wistar rats fed an HFD over a 16-week period. (**A**) Body weight changes of male Wistar rats in each group over 16 weeks. (**B**) The Lee Index and (**C**) body mass index (BMI) at the end of the study. The rats were divided into five groups: normal diet (ND), high-fat diet (HFD), and HFD supplemented with the honey-conjugated HBb-Bps at doses of 200, 500, and 1000 mg/kg body weight. Data are presented as the mean ± SD (*n* = 6). Statistical differences were analyzed using one-way ANOVA followed by post hoc analysis. Significant differences vs. HFD group: * *p* < 0.05, ** *p* < 0.01; significant differences vs. ND group: # *p* < 0.05, ## *p* < 0.01.

The anti-obesity effects of the honey-conjugated HBb-Bps were examined in a high-fat diet (HFD)-induced obese rat model over a 16-week period. Body weight, the Lee Index, and body mass index (BMI) were used as indicators of obesity and metabolic status. No expected or unexpected adverse events, including mortality, were observed in any of the animals during the study. The body weight changes during the experimental period revealed a clear separation between the groups. The rats in the HFD group exhibited continuous and pronounced weight gain from week 4 onwards, reaching significantly higher weights than the normal diet (ND) group by week 16 (Figure 2A). In contrast, supplementation with honey-conjugated HBb-Bps at all tested doses (200, 500, and 1000 mg/kg body weight) resulted in a dose-dependent reduction in weight gain, with the 1000 mg/kg group showing the lowest final body weight among all the HFD-fed rats.

Evaluation of the Lee Index values (Figure 2B) showed a significant increase in the HFD group compared to the ND group, confirming the development of visceral adiposity. Treatment with the conjugated HBb-Bps led to a significant decrease in the Lee Index, particularly in the HBb-500 and HBb-1000 groups. Similarly, the BMI values (Figure 2C) were significantly reduced by the conjugated peptides, with the highest dose (1000 mg/kg) producing a comparable BMI to that of the ND group. These findings suggest a protective effect of the conjugated peptides against diet-induced obesity.

**Figure 3:**
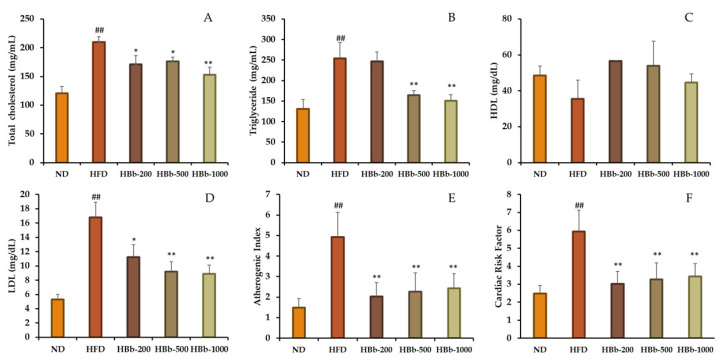
Effects of honey-conjugated HBb-Bps on serum lipid profiles and cardiovascular risk parameters in Wistar rats fed an HFD. (**A**) Total cholesterol, (**B**) triglyceride, (**C**) high-density lipoprotein (HDL), and (**D**) low-density lipoprotein (LDL) levels, (**E**) Atherogenic Index, and (**F**) Cardiac Risk Factor in male Wistar rats after 16 weeks of treatment. The rats were assigned to five groups: normal diet (ND), high-fat diet (HFD), and HFD supplemented with the honey-conjugated HBb-Bps at doses of 200, 500, and 1000 mg/kg body weight. Data are expressed as the mean ± SD (*n* = 6). Statistical analysis was performed using one-way ANOVA with post hoc comparisons. Significant differences vs. HFD group: * *p* < 0.05, ** *p* < 0.01; significant differences vs. ND group: ## *p* < 0.01.

The effects of the honey-conjugated HBb-Bps on lipid metabolism and cardiovascular risk were evaluated in HFD-fed Wistar rats after 16 weeks of the dietary intervention. The HFD group exhibited significantly elevated total cholesterol (210.00 ± 9.46 mg/dL), triglyceride (254.34 ± 37.83 mg/dL), and LDL (16.79 ± 2.11 mg/dL) levels compared to the ND group (120.47 ± 11.81, 130.75 ± 22.87, and 5.28 ± 0.74 mg/dL, respectively), indicating diet-induced dyslipidemia (Figure 3A,B,D). Supplementation with the honey-conjugated HBb-Bps resulted in dose-dependent reductions in these parameters. The group that received 1000 mg/kg showed the most prominent effect, with a reduction in the total cholesterol level to 153.00 ± 13.23 mg/dL and triglyceride level to 150.34 ± 18.87 mg/dL, both significantly lower than the HFD group (*p* < 0.01). Similarly, the LDL levels in this group dropped to 8.86 ± 1.24 mg/dL, nearly 50% below that of the HFD controls. The HDL levels were reduced in the HFD group (35.41 ± 10.51 mg/dL), indicating impaired lipid metabolism. In contrast, all the groups treated with the honey-conjugated HBb-Bps showed increased HDL concentrations. The most notable elevation was observed in the HBb-200 group, which reached 56.63 ± 0.28 mg/dL. However, these differences were not statistically significant compared to the HFD group (Figure 3C). More importantly, the Atherogenic Index and Cardiac Risk Factor values were markedly elevated in the HFD group (4.93 ± 1.20 and 5.93 ± 1.32, respectively), but significantly decreased in all the treatment groups (Figure 3E,F). The HBb-1000 group demonstrated the greatest risk reduction, with values of 2.42 ± 0.72 for the Atherogenic Index and 3.42 ± 1.04 for Cardiac Risk Factor (*p* < 0.01). These findings collectively suggest that honey-conjugated HBb-Bps exert protective effects against HFD-induced lipid abnormalities and may mitigate cardiovascular risk through modulating serum lipid profiles.

**Figure 4:**
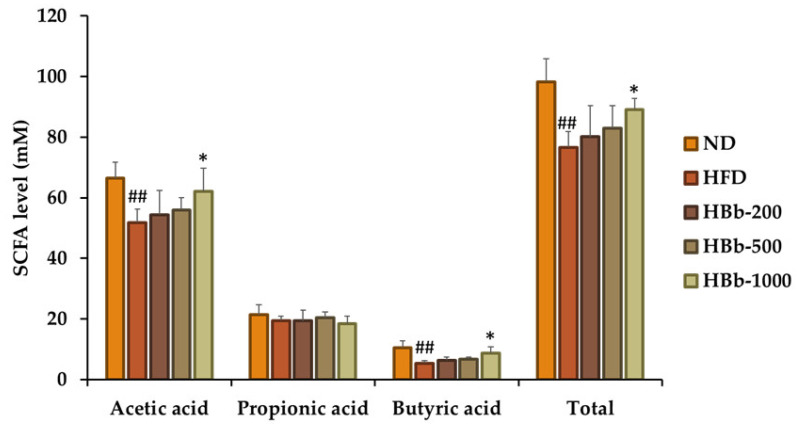
Fecal short-chain fatty acid (SCFA) profiles in Wistar rats fed an HFD and honey-conjugated HBb-Bps. Levels of acetic acid, propionic acid, butyric acid, and total SCFAs in the feces of male Wistar rats after 16 weeks of dietary intervention. The rats were assigned to five groups: normal diet (ND), high-fat diet (HFD), and HFD supplemented with the honey-conjugated HBb-Bps at doses of 200, 500, and 1000 mg/kg body weight. Data are presented as the mean ± SD (*n* = 6). Statistical analysis was performed using one-way ANOVA followed by post hoc comparisons. Significant differences vs. HFD group: * *p* < 0.05; significant differences vs. ND group: ## *p* < 0.01.

The fecal short-chain fatty acid (SCFA) profiles were analyzed to determine the effects of the honey-conjugated HBb-Bps on microbial fermentation and gut metabolic activity in the HFD-fed Wistar rats. The HFD group showed markedly lower levels of key short-chain fatty acids (SCFAs) compared to the normal diet (ND) group. Specifically, the acetic acid and butyric acid levels decreased to 51.86 ± 4.36 mM and 5.32 ± 0.93 mM, respectively, in contrast to the ND group’s values of 66.43 ± 5.24 and 10.56 ± 2.20 mM. The total SCFA concentration also declined from 98.31 ± 7.62 mM in the ND group to 76.63 ± 5.14 mM in the HFD group. These reductions indicate a disruption in microbial SCFA production under obesogenic dietary conditions (Figure 4). A recovery in SCFA concentrations was observed following treatment with the honey-conjugated HBb-Bps. In particular, the acetic acid levels modestly increased in all the treatment groups. The HBb-500 group exhibited the highest acetic acid concentration (55.95 ± 3.96 mM), which was significantly higher than that of the HFD group (*p* < 0.05). Notably, the butyric acid levels increased in a dose-dependent manner, with the HBb-1000 group achieving a level of 8.69 ± 2.10 mM, which is significantly higher than that of the HFD group (*p* < 0.05) and approaches the level seen in the ND rats. Although the propionic acid levels remained relatively stable across all the groups, the total SCFA concentrations were higher in all the treatment groups, with HBb-500 showing the greatest restoration (83.02 ± 7.32 mM; *p* < 0.05 vs. HFD). These results suggest that honey-conjugated HBb-Bps can enhance gut microbial SCFA production, particularly butyrate, which may contribute to the metabolic and anti-inflammatory benefits observed in the rat model.

## 4. Discussion

This study aimed to extend the previous findings on honeybee brood-derived biopeptides, which were shown to exhibit health-promoting properties, particularly in terms of their ability to enhance the growth of beneficial probiotic strains following Maillard conjugation with sugars or honey. To further substantiate their prebiotic potential, we evaluated their resistance to gastrointestinal digestion and examined their physiological effects in a rat model. These investigations provide critical evidence supporting their functional stability and bioactivity, reinforcing their promise as novel prebiotic candidates for future food and nutraceutical applications.

The functional efficacy of prebiotics relies heavily on their ability to resist degradation in the upper gastrointestinal (GI) tract, thereby allowing them to reach the colon intact where they can exert selective stimulatory effects on beneficial gut microbiota [16,17]. According to the International Scientific Association for Probiotics and Prebiotics (ISAPP), one of the critical criteria for a compound to be classified as a prebiotic is its resistance to digestion in the mouth, stomach, and small intestine [22]. Simulated GI digestion systems have become widely adopted as standardized models to evaluate the digestibility and survival of potential prebiotic compounds before conducting in vivo studies [23,24]. Previous studies have demonstrated that protein hydrolysates and specific bioactive peptides may exhibit partial resistance to GI enzymes, but their survival can vary significantly depending on their amino acid composition and structural modifications [25].

In this study, the honey-conjugated HBb-Bps displayed markedly improved digestion resistance across all the simulated GI phases compared to the native HBb-Bps. Most notably, the extent of hydrolysis in the gastric phase was reduced from 36.41% in the native HBb-Bps to only 0.36% in the honey-conjugated forms, with a high residual post-digestion content that was comparable to that of inulin. This enhanced resistance can be attributed to multiple complementary mechanisms. First, the Maillard reaction during conjugation generated glycated structures and melanoidin-like complexes, both of which are known to improve proteolytic resistance and exhibit prebiotic properties [26,27]. Previous studies found that glycation of ovalbumin by α-dicarbonyl compounds significantly impaired proteolytic digestibility and altered their cleavage patterns [28]. In addition, the covalent bonding between peptide amino groups and reducing sugars likely masks protease cleavage sites, thereby limiting enzyme accessibility [29]. Second, the presence of bulky sugar moieties from honey may provide steric hindrance, further obstructing enzymatic attack on the peptide backbone [30]. Third, our previous work [9] demonstrated that honey conjugation altered the sugar composition, amino acid profile, peptide molecular weight distribution, and degree of glycation of HBb-Bps, resulting in greater molecular heterogeneity and steric protection, features that can slow proteolysis. Fourth, Maillard conjugation can induce conformational changes, such as an increase in the β-sheet content and the formation of protein–carbohydrate aggregates, which enhance structural stability under acidic gastric conditions [31]. Together with the limited degradation observed in the oral and intestinal phases, these results suggest that they are stable as they pass through the digestive tract. These data indicate that honey conjugation not only preserves the structural integrity of biopeptides but also improves their likelihood of surviving upper gastrointestinal transit, thereby enhancing their potential functionality as prebiotic agents. Future studies should employ targeted experimental approaches, such as protease cleavage site mapping to identify digestion-resistant regions, and glycation site characterization via mass spectrometry, to validate the proposed mechanisms underlying the enhanced gastrointestinal stability of honey-conjugated HBb-Bps.

The antioxidant activities of both the HBb-Bps and honey-conjugated HBb-Bps were clearly demonstrated, with the conjugated form showing significantly greater radical-scavenging capacity in both the DPPH and ABTS assays. In our previous study [9], ferric reducing antioxidant power (FRAP) assays were also performed, and the results closely paralleled those of the DPPH and ABTS tests, further confirming that honey conjugation consistently improved the antioxidant capacity of HBb-Bps. This enhancement may be attributed to the formation of Maillard reaction products (MRPs), particularly melanoidin-like structures, which have been reported to possess strong antioxidant properties due to their ability to donate electrons and scavenge free radicals [32,33]. These results are consistent with our previous findings, which demonstrated that glycation of honeybee brood peptides through the Maillard reaction significantly enhanced their antioxidative potential via the formation of bioactive conjugates and by increasing the phenolic and flavonoid contents [9]. In that earlier study, we also demonstrated that using the moist–dry heating process for honey–peptide conjugation altered multiple chemical characteristics, including a broader range of peptide sizes and a higher glycation index compared to the native form, changes that are likely to enhance both their physicochemical stability and bioactivity. Upon simulated gastrointestinal digestion, both peptide types exhibited reduced antioxidant activity, likely due to enzymatic degradation or conformational changes that disrupted redox-active sites. However, the reduction was less pronounced in the honey-conjugated HBb-Bps, indicating improved structural stability and resistance to degradation. Similar trends have been observed in other glycated protein hydrolysates, where glycation enhances antioxidative stability under digestive conditions [34,35]. Such outcomes imply that conjugation with honey not only confers structural protection against enzymatic degradation, but it also contributes to the retention of antioxidant functionality under simulated gastrointestinal conditions.

Our findings align with those of previous studies on Maillard-conjugated peptides from various protein sources, which consistently report enhanced antioxidant activity, improved gastrointestinal stability, and potential prebiotic effects. For instance, soy protein hydrolysates conjugated via the Maillard reaction with reducing sugars (e.g., mannose and allulose) demonstrated a markedly improved antioxidant capacity, better digestibility, and enhanced sensory attributes such as umami and caramel-like flavors [36,37]. In addition, research on insect protein hydrolysates has shown that Maillard conjugation significantly enhances functional properties. Maillard reaction of cricket and mealworm hydrolysates resulted in more complex savory flavor profiles with a higher number of odor-active compounds [38]. Another study on black cricket (*Gryllus assimilis*) protein hydrolysates indicated that Maillard reaction improved their antioxidant and potentially antihypertensive activities [39]. Collectively, these findings align with our current results, situating honey-conjugated HBb-Bps within a broader pattern of enhanced bioactivity following Maillard conjugation. However, honey’s unique composition, which is rich in reducing sugars, phenolics, and oligosaccharides, may offer synergistic benefits beyond what is achievable with isolated sugars or other proteins.

High-fat diet (HFD)-induced obesity is a widely used experimental model for mimicking metabolic syndrome in rodents, as it effectively promotes visceral fat accumulation, insulin resistance, and dyslipidemia, the key features of human obesity and metabolic dysfunction [40,41]. Emerging evidence has highlighted the crucial role of gut microbiota in regulating host metabolism. Dysbiosis, which involves a reduction in microbial diversity and an imbalance in the Firmicutes to Bacteroidetes ratio, has been strongly associated with various metabolic disorders [42]. Prebiotics, defined as selectively fermented ingredients that induce beneficial changes in gut microbiota composition and activity, have been shown to counteract such imbalances [43]. By stimulating the growth of beneficial bacteria such as Bifidobacteria and Lactobacilli, prebiotics enhance the microbial production of short-chain fatty acids (SCFAs), including acetate, propionate, and butyrate, which serve as signaling molecules that can regulate energy homeostasis, improve gut barrier integrity, and reduce systemic inflammation [44,45]. These SCFAs also modulate metabolic parameters by influencing glucose and lipid metabolism through G-protein-coupled receptor activation and histone deacetylase inhibition, thereby inducing anti-obesity and insulin-sensitizing effects [46]. Therefore, the ability of functional ingredients to promote SCFA production and support gut microbial balance represents a promising strategy for ameliorating diet-induced metabolic syndrome.

The selection of the doses used in this study was guided by preliminary safety data. An acute oral toxicity evaluation of the HBb-Bps, conducted according to OECD guideline 420, demonstrated no mortality or adverse clinical signs at a single dose of 2000 mg/kg body weight. Thus, for the 16-week high-fat diet intervention in the present study, lower doses of 200, 500, and 1000 mg/kg were chosen to remain well below the no-observed-adverse-effect level, thereby minimizing potential long-term toxicity risks. Oral administration of the honey-conjugated HBb-Bps led to significant improvements in body weight regulation, adiposity indices, and lipid profiles, highlighting their therapeutic potential in managing metabolic syndrome. These outcomes agree with previous reports on bioactive peptides derived from food proteins such as soy, milk casein, fish collagen, and egg white, which have shown similar anti-obesity and hypolipidemic effects in rodent models through modulating lipid metabolism, suppressing adipogenesis, and enhancing energy expenditure [47,48,49]. The observed increases in fecal acetate and butyrate levels suggest an additional gut microbiota-mediated mechanism, as SCFAs are known to influence satiety, lipid metabolism, and inflammation [50,51]. Honey-derived oligosaccharides likely acted as the primary driver for SCFA elevation by serving as fermentable substrates for beneficial bacteria, while Maillard reaction products and glycated peptides contributed to sustained substrate availability and structural stability in the colon. This combination likely enhanced the peptide’s prebiotic functionality and digestive stability, thereby increasing the microbial fermentation capacity. The resulting synergistic enhancement of SCFA production could in turn promote beneficial effects on host metabolism, including improved lipid regulation, reduced adiposity, and increased energy expenditure.

Although this study did not directly examine the changes in gut microbiota composition, the alterations in SCFA concentrations provided indirect evidence of the microbial activity response to the treatment. SCFAs, including acetate, propionate, and butyrate, are key microbial metabolites that are known to influence host energy homeostasis, gut barrier integrity, and systemic inflammation [44,52]. In the high-fat diet-induced obese rats, SCFA levels were markedly reduced, consistent with the known link between dysbiosis and an impaired microbial fermentation capacity. Notably, supplementation with the honey-conjugated HBb-Bps significantly increased the acetic acid and total SCFA levels, particularly in the HBb-500 group, while the butyrate concentrations also showed notable improvements. These findings suggest that the conjugated peptides may have influenced the microbial fermentation activity. However, we did not confirm that selective fermentation by beneficial microbes occurred. The absence of metagenomic or 16S rRNA profiling, mechanistic experiments, and long-term safety or toxicity evaluations remains a limitation, especially for potential human application. Future studies should include these approaches, along with targeted fermentation assays, to validate the microbiota shifts and identify the taxa responsible for SCFA generation. These data would strengthen our understanding of the underlying prebiotic mechanisms and support the development of honey–peptide conjugates as multifunctional dietary interventions targeting both gut microbiota and host metabolism.

## 5. Conclusions

Honey-conjugated honeybee brood biopeptides (HBb-Bps) demonstrated improved digestive stability, antioxidant activity, and favorable biological effects compared to native HBb-Bps in a HFD-induced rat model. In this preclinical setting, the conjugated peptides were associated with reduced weight gain, improved lipid profiles, and lower cardiovascular risk indices. They also increased short-chain fatty acid production, suggesting potential modulation of gut microbiota fermentation. These findings provide preliminary evidence supporting the potential of honey-conjugated HBb-Bps as functional ingredients for metabolic health management. Future work should incorporate metagenomic or 16S rRNA sequencing to confirm the microbial shifts, alongside comprehensive safety evaluations, regulatory assessments, and longer-term safety assessments, regulatory evaluations, and extended intervention trials to validate their applicability in human nutrition.

## Figures and Tables

**Table 1:** Degree of hydrolysis and residual post-digestion content after simulated gastrointestinal digestion of inulin, HBb-Bps, and honey-conjugated HBb-Bps.

Sample	Degree of Hydrolysis (%)			Residual Post-Digestion Content (%)
	Mouth	Stomach	Small Intestine	
Inulin	0.04 ± 0.01	1.60 ± 0.23	15.85 ± 1.27	82.51 ± 3.14
HBb-Bps	4.13 ± 0.34	36.41 ± 2.14	12.46 ± 1.18	46.99 ± 2.44
Honey-conjugated HBb-Bps	0.27 ± 0.08	0.36 ± 0.10	13.25 ± 3.14	86.12 ± 4.01

**Table 2:** Antioxidant activity of HBb-Bps and honey-conjugated HBb-Bps before and after digestion.

Assay	Time Point	% Radical-Scavenging Activity		% Difference Between HBb-Bps and Honey-Conjugated HBb-Bps	Exact *p*-Value Between HBb-Bps and Honey-Conjugated HBb-Bps	Effect Size
		HBb-Bps	Honey-Conjugated HBb-Bps			
DPPH	Before digestion	31.04 ± 2.41	43.28 ± 4.10	39.43	0.0021	2.91
	After digestion	15.36 ± 3.45	25.61 ± 2.39	66.73	0.0054	2.76
ABTS	Before digestion	54.21 ± 5.34	70.54 ± 3.48	30.12	0.0315	2.90
	After digestion	32.14 ± 4.25	60.32 ± 4.27	87.68	0.0104	5.90

% Radical-Scavenging Activity (%RSA) represents the percentage reduction in DPPH or ABTS radicals by the sample compared to a control. Values are presented as the mean ± SD from three independent experiments (*n* = 3). Statistical significance between the HBb-Bps and honey-conjugated HBb-Bps was determined using one-way ANOVA followed by Tukey’s post hoc test, with the exact *p*-values and effect sizes (Hedges’ g) indicated. Differences between groups were considered significant if *p* < 0.05.

## Data Availability

The data supporting the findings of this study are available within the article.
